# Editorial: Mathematical Modeling of the Immune System in Homeostasis, Infection and Disease

**DOI:** 10.3389/fimmu.2019.02944

**Published:** 2020-01-08

**Authors:** Gennady Bocharov, Vitaly Volpert, Burkhard Ludewig, Andreas Meyerhans

**Affiliations:** ^1^Marchuk Institute of Numerical Mathematics of the Russian Academy of Sciences, Moscow, Russia; ^2^Institute for Personalized Medicine, Sechenov First Moscow State Medical University, Moscow, Russia; ^3^Institut Camille Jordan, Université Lyon 1, Villeurbanne, France; ^4^INRIA Team Dracula, INRIA Lyon La Doua, Villeurbanne, France; ^5^Peoples' Friendship University of Russia (RUDN University), Moscow, Russia; ^6^Institute of Immunobiology, Kantonsspital St. Gallen, St. Gallen, Switzerland; ^7^Infection Biology Laboratory, Department of Experimental and Health Sciences, Universitat Pompeu Fabra, Barcelona, Spain; ^8^Institució Catalana de Recerca i Estudis Avançats (ICREA), Barcelona, Spain

**Keywords:** immune system, mathematical modeling, complexity, multiscale (MS) modeling, spatial organization, regulation, immune-related diseases

The immune system is a dynamic and multi-level biological system that protects host organisms against invading pathogens and tumor development, and plays an active role in tissue homeostasis and organ regeneration. As such, it needs to respond to a vast diversity of threats while minimizing damage to its own cells and organs. To perform this task, it is organized as a tightly regulated, hierarchically controlled and spatially distributed network with soluble and cellular components. Recent technical advances including life-imaging, multi-color phenotyping, and “omics” technologies provide with an unprecedented level of detail of a functional immune system. Since mathematics is the universal language for expressing causal and functional relationships between observations, it is natural to use mathematical tools for mechanistically describing immune system dynamics and functioning. However, as both the immune system's complexity and experimental data sets are huge, it is a substantial challenge to connect these in a mechanistic way. The major problems in this respect are known as “curse of dimensionality” and “combinatorial explosion.” The mainstream research in this field is still based on low-resolution models that often provide only limited descriptions of individual immune system components and their interactions after external stimulations. Shifting this simplistic perception of the immune system to a dynamic, multi-level, and spatially resolved system description with molecular and cellular networks is daunting and requires the combination of a solid understanding of the underlying systems biology with the application of appropriate mathematical methodologies. This may ultimately improve the biological relevance of the generated models and contribute to a better mechanistic understanding of immune system functioning as well as making biologically and clinically relevant predictions for diagnosis and treatment of human diseases.

The aim of this Research Topic was to present current state-of-the-art research on using mathematically driven exploration of the complexity of the immune system. A series of articles were collected, giving a comprehensive overview of conceptual frameworks and emerging topics including the “spatial organization of the immune system” and “multifactorial immune-related diseases.”

In his conceptual review, Grossman focused on the “smart surveillance” theory of how T cells individually and collectively respond to self- and foreign antigens depending on contextual parameters. He highlighted that the physiological messages to cells are encoded not only in the biochemical connections of sets of signaling molecules to the cellular machinery but also in their magnitude, kinetics, and time and space contingencies. The “dynamic tuning hypothesis” is a central component of his theory and sets the ground for further theoretical and experimental exploration of immune tolerance, homeostasis, and diversity. Moreover, Grossman used his conceptual postulates to discuss conflicting models of HIV pathogenesis. Castiglione et al. addressed the underlying mechanism of cross-reactive immune responses and antigenic sin that may be beneficial, neutral, or detrimental for the host. They studied the relationship of clonal dominance with memory cell attrition with an agent-based model. They propose that attrition could serve as a curbing mechanism for the memory-anti-naive phenomenon.

Several studies describe novel modeling tools and their applications. Lanzarotti et al. developed a model for the prediction of cognate T cell receptor (TCR) targets. It is based on the similarity to a database of TCRs with known targets and may have important implications for the rational design of T cell-based therapies. With their time-resolved experimental data of splenic transcriptomes from mice infected with the lymphocytic choriomeningitis virus (LCMV), Pedragosa et al. addressed the problem of linking gene expression changes from whole tissue with immune cell dynamics. To this end, they combined weighted gene co-expression network analysis—with digital cell quantifier—providing a novel approach to bridge the genomic with the cellular level during antiviral immune responses. Meier-Schellersheim et al. discuss how mechanistic rule-based modeling can be used to test immunological hypotheses through quantitative simulations. They considered as an example G-protein-coupled receptor signaling that is utilized by cells to respond to a wide range of extracellular stimuli and explore the cross-talk of multiple cytokine pathways, thereby providing basis for deriving cell population behavior from single-cell models and bridging a current scale gap. Finally, Enciso et al. demonstrated how discrete dynamic models can be transformed to continuous dynamic models using Fuzzy logic. This approach enables a better description of growth and differentiation of T lymphocytes in various microenvironments.

The consequences of an uneven partitioning of molecular contents on cell fate regulation were studied by Girel et al. They introduced a multi-scale mathematical model of CD8 T cell responses in lymph nodes and showed that the degree of unevenness of molecular partitioning affects the outcome of the immune response and memory cell generation. Huang et al. considered virus and interferon spread within an infected host as two competing processes and analyzed a well-mixed vs. a spatially segregated scenario. They defined the conditions under which the interferon response works most effectively and suppresses the infection.

A series of six publications considered the architecture and functioning of lymph nodes within an immune response. Novkovic et al. reviewed available computational lymph node models with the focus on the structure and organization of stromal cells. The authors pointed out that hybrid- and multi-scale models in combination with high-resolution imaging will be important to unravel the complex immune mechanisms that are initiated in lymph nodes. The study by Moses et al. is focused on the definition of rules specifying the search strategies of T cells for antigen. They discovered striking similarities between the strategies ant colonies use to forage and the immune cells use to find pathogens. The strategies are based on a variety of search behaviors including directional movement using chemokine gradients, random motion using correlated random walk, and movement along physical networks. Kalogiros et al. developed a mathematical framework to characterize spatio-temporal chemokine gradient formation. With their Bayesian parameter inference approach, they provided a building block for subsequent multi-scale modeling. Azarov et al. developed an agent-based model to investigate the role of T cell-dendritic cell (DC) chemoattraction in T cell priming in the lymph node. They stressed that the balance of naive and activated antigen-specific T cells that are both chemotactically attracted to the neighborhood of DCs determine the overall amplitude of the specific T cell response. Grebennikov et al. developed a physics-based model of T cell motility in lymph nodes. The cell dynamics is determined by a superposition of autonomous locomotion, intercellular interactions, and viscous dumping. The model was then used to predict the required CD8 T cell frequencies necessary to detect HIV-infected cells before they start releasing virus particles. McDaniel and Ganusov studied lymphocyte recirculation in sheep. With a series of mathematical models, they estimated the distribution of residence times in ovine lymph nodes.

Finally, six publications addressed various aspects of multi-factorial immune-related phenomena and diseases. Presbitero et al. described the role of alkaline phosphatase (AP) during cardiac surgery. They developed a mathematical model of systemic inflammation and suggested that supplemented AP provides a patient benefit by inducing liver-type tissue non-specific AP production. Coulibaly et al. formulated a mathematical model that describes the molecular mechanisms involved in the IL-15-induced signaling cascade of the hypoxia-inducible factor 1α (HIF-1α) pathway in natural killer cells. In combination with experimental work, they identified mammalian target of rapamycin (mTOR), the nuclear factor-kB (NF-kB), and the signal transducer and activator of transcription 3 (STAT3) as central regulators of HIF-1α accumulation. Benchaib et al. studied the interaction between cancer and immune cells in the lymph node. They delineated with mathematical models the conditions for the three possible outcomes, namely, tumor elimination, equilibrium, and tumor evasion. The study of Blickensdorf et al. compared fungal infections with *Aspergillus fumigatus* in murine and human lungs. They analyzed the spatial infection dynamics with a hybrid agent-based model that accounts for the specific lung physiologies. Infections are more efficiently cleared in mice due to their smaller alveolar surface areas. Peskov et al. reviewed the state of the art of quantitative systems models describing tumor and immune system interactions and discussed approaches for biomarker identification. Finally, Nikolaev et al. studied fundamental interactions between a pathogen with a tumor. Their work is based on the recent finding that an acute influenza infection in the lung promotes melanoma growth in the dermis of mice. Using models of complex intracellular biochemical reaction networks, they analyzed virus-specific and melanoma-specific CD8 T cells in the lung. They proposed that the observed melanoma growth results from sequestering of tumor-specific effector cells in the lung due to their loss of motility via PD-1 interactions. In contrast, virus-specific T cells remain functional and clear the influenza infection since they adapt to the strong stimulation by their cognate antigen locally.

Collectively, this Research Topic highlighted the ongoing attempts to quantitatively describe and mechanistically understand the complex interactions inherent in immune system functioning during normal conditions and in disease. While far from providing a complete view, important mathematical elements of systems immunology are emerging that are based on genuine collaborations between experimentalists and applied mathematicians. Only with such multi-disciplinary efforts will we be able to enrich immunological research with analytical and predictive modeling tools that complement the impressive advances in observational technologies. This area of research is and will continue to flourish.

## Author Contributions

All authors listed have made a substantial, direct and intellectual contribution to the work, and approved it for publication.

### Conflict of Interest

The authors declare that the research was conducted in the absence of any commercial or financial relationships that could be construed as a potential conflict of interest.

